# Poverty and inequality in real-world schizophrenia: a national study

**DOI:** 10.3389/fpubh.2023.1182441

**Published:** 2023-10-31

**Authors:** Guillaume B. Fond, Dong Keon Yon, Bach Tran, Jasmina Mallet, Mathieu Urbach, Sylvain Leignier, Romain Rey, David Misdrahi, Pierre-Michel Llorca, Franck Schürhoff, Fabrice Berna, Laurent Boyer

**Affiliations:** ^1^Fondation FondaMental, Créteil, France; ^2^AP-HM, Aix-Marseille Univ, Faculté de Médecine – Secteur Timone, EA 3279: CEReSS – Centre d’Etude et de Recherche sur les Services de Santé et la Qualité de vie, Marseille, France; ^3^Department of Pediatrics, Kyung Hee University College of Medicine, Seoul, Republic of Korea; ^4^Center for Digital Health, Medical Science Research Institute, Kyung Hee University Medical Center, Kyung Hee University College of Medicine, Seoul, Republic of Korea; ^5^Research Centre on Health Services and Quality of Life, Aix Marseille University, Marseille, France; ^6^Department of Psychiatry, Université de Paris, AP-HP, Louis Mourier Hospital, INSERM UMR 1266, Paris, France; ^7^Service Universitaire de Psychiatrie d’Adultes et d’Addictologie, Centre Hospitalier de Versailles, Paris, France; ^8^Centre Référent de Réhabilitation Psychosociale, CH Alpes Isère, Grenoble, France; ^9^INSERM U1028, CNRS UMR5292, Centre de Recherche en Neurosciences de Lyon, Université Claude Bernard Lyon 1, Equipe PSYR2, Centre Hospitalier Le Vinatier, Bron Cedex, France; ^10^Pôle de Psychiatrie Générale et Universitaire, Centre Hospitalier Charles Perrens, Université de Bordeaux, Bordeaux, France; ^11^CNRS UMR 5287-INCIA, Bordeaux, France; ^12^Université Clermont Auvergne, CMP-B CHU, CNRS, Clermont Auvergne INP, Institut Pascal, F-63000 Clermont-Ferrand, France; ^13^Université Paris Est Creteil (UPEC), AP-HP, Hôpitaux Universitaires «H. Mondor», DMU IMPACT, INSERM, IMRB, Translational Neuropsychiatry, Fondation FondaMental, Creteil, France; ^14^Hôpitaux Universitaires de Strasbourg, Université de Strasbourg, INSERM U1114, Fédération de Médecine Translationnelle de Strasbourg, Strasbourg, France

**Keywords:** schizophrenia, psychotic disorders, mental health, poverty, inequality, public health, psychiatry, quality of life

## Abstract

**Background:**

Schizophrenia has high socioeconomic impact among severe psychiatric disorders.

**Aims:**

To explore clinician-reported and patient-reported inequities between patients under the poverty threshold vs. the others.

**Method:**

916 patients consecutively recruited in 10 national centers received a comprehensive standardized evaluation of illness severity, addictions and patient-reported outcomes.

**Results:**

739 (80.7%) of the patients were classified in the poverty group. This group had poorer objective illness outcomes (lower positive, negative, cognitive, excitement/aggressive and self-neglect symptoms and lifetime history of planned suicide) in multivariate analyses. While they had similar access to treatments and psychotherapy, they had lower access to socially useful activities, couple’s life, housing and parenthood. They had also more disturbed metabolic parameters. On the contrary, the poverty group reported better self-esteem. No significant difference for depression, risky health behavior including addictions and sedentary behavior was found.

**Interpretation:**

The equity in access to care is attributed to the French social system. However, mental and physical health remain poorer in these patients, and they still experience poor access to social roles independently of illness severity and despite healthcare interventions. These patients may have paradoxically better self-esteem due to decreased contact with society and therefore lower stigma exposure (especially at work). Schizophrenia presents itself as a distinct impoverished population concerning health-related outcomes and social integration, warranting focus in public health initiatives and improved treatment, including tailored interventions, collaborative care models, accessible mental health services, housing support, vocational training and employment support, community integration, education and awareness, research and data collection, culturally competent approaches, and long-term support.

## Introduction

The 100% coverage health insurance system in France was established in 1945 after World War II to eliminate the disparities in healthcare access stemming from financial inequalities. “Inequities” refer to disparities in access to healthcare services and variations in health outcomes among patients with schizophrenia. As of now, the evidence supporting the effectiveness of social protection policies in addressing health inequalities remains limited. A majority of the published data has primarily focused on the Western systems, particularly those in the US and UK (1).

Among all severe mental disorders, schizophrenia exhibits the most pronounced connection with poverty, attributed to several clinical factors (including delusions, negative symptoms, and poor insight into illness) as well as social stigma, which hampers employment opportunities. The onset age of schizophrenia typically falls between 18 and 25 years for the majority of patients, a critical period for pursuing education and entering the workforce. As of 2021 in France, individuals unemployed due to schizophrenia can receive a disability allowance of 903 euros, while the poverty line is estimated at 1063 euros per month in the country. Moreover, all patients receive full reimbursement for pharmacological treatments (2). A recent study has revealed that schizophrenia exhibited the highest prevalence among all mental health disorders in sex workers, prisoners, and individuals with substance use disorders (3).

A study conducted within the Danish population has revealed that a low parental income was associated with an increased risk of schizophrenia onset in their offspring (4). While medico-economic studies of schizophrenia have primarily concentrated on its impact on caregivers and society, there is limited understanding regarding the influence of poverty on the clinical outcomes of the disease. Our team has published the findings of the French “Housing First” 2 years follow-up program, demonstrating that providing housing to homeless individuals with schizophrenia enhanced their access to care and subsequently improved clinical outcomes (5–8). A recent study conducted in low-to-middle-income countries has indicated that poverty was linked to positive symptoms in individuals with schizophrenia (9). While stigma is not a direct health outcome, it can influence treatment adherence and overall well-being among individuals with schizophrenia (10). In summary, while it is anticipated that individuals with more severe symptoms may experience lower income due to the disability caused by their illness, the specific clinical traits associated with poverty remain undetermined up to the present time. This also applies to their connection with various aspects of functioning, quality of life, and their access to care, including both pharmacological treatments and psychotherapy.

The FACE-SZ (FondaMental Academic Center of Expertise for Schizophrenia) cohort has been established with funding from the French Ministry of Research, aiming to create a national cohort of outpatients with schizophrenia. This cohort is still in progress and has already generated clinical recommendations aimed at enhancing the provision of mental health care for schizophrenia outpatients (11). However, the factors associated with poverty have not been explored up to this point.

The objective of this study was to identify the factors associated with poverty among the participants of FACE-SZ at the time of their enrollment in the cohort. We hypothesized that individuals with lower income would exhibit a lower education level, a higher comorbidity of substance use, more severe symptoms of illness, increased somatic comorbidities, impaired functioning, and diminished quality of life.

## Population and methods

### Design

This study is a cross-sectional observational study. All patients referred between January 2015 and December 2018 to the ten Schizophrenia Expert Centers located in Bordeaux, Clermont-Ferrand, Colombes, Créteil, Grenoble, Lyon, Marseille, Montpellier, Strasbourg, and Versailles^1^ were consecutively included (11, 12). These expert centres cover the entire French territory. All patients were referred by their general practitioner or psychiatrist, and they received a comprehensive evaluation report along with personalized intervention recommendations.

### Inclusion and exclusion criteria

The current study involved clinically stabilized patients who met the following inclusion criteria: a DSM-5 diagnosis of schizophrenia or schizoaffective disorder, and no hospitalization or alterations in treatment within the 8 weeks preceding the assessment (13).

Two skilled psychiatrists from the Schizophrenia Expert Centers network validated the diagnosis using the Structured Clinical Interview for Mental Disorders (SCID-1.0) (13).

Patients with psychiatric comorbidities, with the exception of major depression, anxiety disorders, eating disorders, and addictions, as well as those who were not proficient in French, were excluded from the study.

### Collected data

#### Poverty group definition

In alignment with the National Institute of Statistics and Economic Studies, the monetary poverty line is defined as 60% of the median standard of living within the population. For the year 2018, this value was estimated at 1063 euros (14). The FACE-SZ dataset records monthly income as a binary variable (< or ≥1,000 euros/month), which was selected as a proxy for defining poverty when the dataset was established in 2010 (15).

Since schizophrenia typically emerges between the ages of 18 and 25, its influence on employment usually manifests early in one’s professional life. Despite the cross-sectional design of our analyses, these variables were thus considered potential outcomes of poverty, with poverty serving as the explanatory variable (independent variable).

#### Sociodemographics and clinician-rated outcomes

Sociodemographic data were reported: age (continuous), sex (binary), and education level (academic level vs. others, binary). Clinician-rated outcomes were extracted from a comprehensive clinical battery of standardized scales for schizophrenia assessment: Clinical Global Impression scale (CGI) (16), psychotic symptomatology (PANSS total score and positive and negative factors) (continuous) (17), current depressive symptoms (Calgary score, continuous) (18) (binary), insight into illness (Birchwood total score) (continuous) (19), lifetime history of planned suicide (Interview on Suicidal Feelings/ISF) (20), (binary). All evaluators were trained in the FondaMental Schizophrenia Expert Network, with regular training sessions (at least once a year) (11).

Current functioning was evaluated using the Personal and Social Performance scale (PSP) (21), which exclusively encompasses functioning-related items with four subscores (socially useful activities, including work and study; personal and social relationships; self-care; and disturbing and aggressive behaviors). It’s important to note for interpretation that PSP subscores are reversed (higher scores correspond to lower functioning) in contrast to the total score (where a higher score signifies better functioning). Additional binary sociality variables were recorded, including relationship status (being single), living arrangements (living alone), and parenthood.

Tobacco, alcohol, and cannabis use disorders were assessed utilizing the standardized expert center battery, which adheres to the DSM-5 criteria (22).

Access to treatments. The following variables were recorded: usage of second-generation antipsychotics, receiving cognitive behavioral therapy within the last 12 months (binary variables), antipsychotic daily dose calculated using the minimum effective dose method (23) and medication Adherence Rating Scale score (MARS) (24).

#### Caregivers reported outcomes

In certain scenarios, caregivers’ assessments can prove to be more pertinent than clinician-rated evaluations (which are confined to the time of assessment and might involve patients withholding information) or patient-reported assessments (which could be influenced by mental illness and self-evaluation). When available, caregivers completed the Evaluation of Cognitive Processes involved in Disability in Schizophrenia scale (ECPDS) (25) with four domains (neurocognition, motivation, insight/awareness of one’s abilities and limitations, and social cognition/communication abilities and ability to understand other people). Neurocognition evaluates the individual’s cognitive abilities, such as memory, attention, problem-solving, and reasoning. It assesses how well the person can process and use information, which is often impaired in individuals with schizophrenia. Motivation assesses the person’s level of motivation and engagement in daily activities. In schizophrenia, motivational deficits are common, and this domain helps gauge the extent to which motivation impacts the individual’s ability to function effectively. Insight/Awareness of One’s Abilities and Limitations evaluates the person’s insight into their condition and their awareness of their own strengths and limitations. Individuals with schizophrenia may struggle with insight, which can affect their ability to make informed decisions about their treatment and daily life. Social Cognition/Communication Abilities and Ability to Understand Other People focuses on the individual’s social and communication skills, as well as their ability to understand and interpret the intentions and emotions of others. Impairments in social cognition are common in schizophrenia and can impact social interactions and relationships. An individual with schizophrenia may experience significant memory deficits that can make it challenging for her/him to remember and follow through with daily tasks and responsibilities, such as managing medications or maintaining employment. In this case, the cognitive problems (memory deficits) are involved in the disability (difficulty in performing daily functions).

The ECPDS scale is designed to provide a comprehensive assessment of these four domains, allowing healthcare professionals to better understand the specific cognitive and functional challenges faced by individuals with schizophrenia. By assessing these areas, clinicians can tailor treatment plans and interventions to address the unique needs of each individual, ultimately improving their quality of life and functioning.

#### Physical health

Physical activity was self-reported by the addition of the weekly duration of moderate and intense physical activity according to the World Health Organization (26). High blood pressure and the history of coronary heart disease were extracted from medical records. Body Mass Index (BMI in kg/m^2^) and abdominal circumference (in centimeters) were measured at the expert center by a trained nurse. Routine blood samples were utilized for measuring Low-Density Lipoprotein-Cholesterol (LDL-C) (g/L), High-Density Lipoprotein-Cholesterol (HDL-C) (g/L), triglyceridemia (g/L), fasting glucose (mM), high sensitivity C Reactive Protein (hs-CRP) (mg/L), and 25-hydroxy-vitamin D3 (cholecalciferol) (nM).

#### Patient-reported outcomes

Self-reported quality of life was assessed through a SZ-specific scale, the Schizophrenia – Quality of Life [SQoL (27)]. The SQoL has eight dimensions (physical well-being, psychological well-being, self-esteem, family relationships, friendships, sentimental life, autonomy and resilience). The inclusion of self-esteem in our analysis of inequalities stems from previous research suggesting that self-esteem can significantly impact mental health outcomes and quality of life (28). Self-reported aggressiveness was evaluated with the Buss & Perry Aggression Questionnaire (BPAQ) (29) with 4 subscores (verbal aggressiveness, physical aggressiveness, anger and hostility).

### Ethical considerations

This study adhered to ethical principles for conducting medical research involving human subjects, as stipulated in the WMA Declaration of Helsinki. The assessment protocol was approved by the pertinent ethical review board (CPP-Ile de France IX, January 18th, 2010). To safeguard participant privacy, all data were gathered anonymously. As the study involved data collected during routine care assessments, all participants signed a non-opposition form.

#### Statistical analysis

The study’s continuous variables were presented as means and standard deviations, while categorical variables were displayed as frequency distributions. Given that the study was designed with predefined hypotheses, no correction for multiple comparisons was conducted, in accordance with the rationale provided (30). For each variable that exhibited a significant association with poverty (binary) in univariate analyses with a value of *p* of <0.2, a multivariate model was constructed. All models were adjusted for age, with sex (which was included in the models by default), education level, poverty, and PANSS total score (excluding the symptom variables). Data analysis was performed using SPSS 20.0 software (SPSS Inc., Chicago, IL). All statistical tests were two-tailed, with a significance level (*α*) set at 0.05.

## Results

A total of 916 stabilized SZ outpatients (240 women and 676 men) from the FACE-SZ cohort were included in the present study. Among them, 739 (80.7%) reported a monthly income of <1,000 euros and were classified into the poverty group. The factors associated with poverty are detailed in Table 1 (clinician-rated and caregivers-rated outcomes) and Table 2 (patient-reported outcomes).

**Table 1:** Clinician-rated and caregivers-rated factors associated with poverty in schizophrenia.

Variables	Univariate model			Multivariate model	
	Poverty *N* = 739 (80.7%)	No poverty *N* = 177 (19.3%)	Univariate *p* value	Adjusted beta coefficients or adjusted OR	Adjusted *p* value
Clinician-rated outcomes					
Sociodemographic variables, *N* or Mean (% or SD)					
Age (years)	**30.5 (9.1)**	**38.4 (9.1)**	**<0.001**		
Sex (male)	547 (74.0%)	129 (72.9%)	0.757		
Academic level	**278 (37.6%)**	**105 (59.3%)**	**<0.001**		
Current illness severity, *N* or Mean (% or SD)*					
Clinical global impression score (CGI score)*	**4.5 (1.1)**	**3.9 (1.2)**	**<0.001**	**−0.10**	**0.001**
Current psychotic severity (PANSS total score)*	**71.1 (19.6)**	**64.6 (17.1)**	**< 0.001**	**−0.12**	**<0.001**
Positive symptoms (PANSS subscore)*	**9.5 (4.5)**	**8.3 (3.9)**	**0.001**	**−0.10**	**0.007**
Negative symptoms (PANSS subscore)*	**17.5 (6.6)**	**15.5 (6.0)**	**<0.001**	**−0.11**	**0.002**
Disorganization (PANSS subscore)*	**8.1 (3.4)**	**7.2 (3.2)**	**0.001**	**−0.08**	**0.020**
Excitment (PANSS subscore)*	**5.9 (2.5)**	**5.3 (2.1)**	**0.001**	**−0.09**	**0.009**
Depressive symptoms (PANSS subscore)*	7.1 (3.2)	7.6 (3.2)	0.058	0.05	0.144
Depressive symptoms (CDSS total score)	3.9 (4.3)	4.0 (4.0)	0.687		
Planned death - Entire life* (ISF questionnaire)	**272 (37.5%)**	**52 (29.5%)**	**0.048**	**0.60 (0.41; 0.88)**	**0.010**
Insight (BIS total score)*	8.6 (3.0)	9.1 (2.9)	0.087	0.02	0.619
Caregivers-rated outcomes					
ECPDS total score*	**52.4 (14.8)**	**59.4 (12.7)**	**0.001**	**0.19**	**0.002**
Neurocognition* (ECPDS subscore)	**16.9 (5.3)**	**19.4 (4.6)**	**0.001**	**0.20**	**0.001**
Motivation* (ECPDS subscore)	**14.4 (5.6)**	**16.9 (5.0)**	**0.001**	**0.17**	**0.006**
Insight to disability* (ECPDS subscore)	**8.2 (2.7)**	**9.0 (2.3)**	**0.031**	**0.13**	**0.046**
Social cognition (ECPDS subscore)	12.9 (4.0)	13.9 (3.5)	0.092	0.11	0.084
Daily Functioning and sociality, *N* or Mean (% or SD)					
Personal and Social performance (PSP total score)	**50.7 (15.9)**	**61.5 (15.3)**	**<0.001**	**0.18**	**<0.001**
Socially useful activities (PSP subscore)	**0.8 (0.4)**	**0.5 (0.5)**	**<0.001**	**−0.24**	**<0.001**
Personal and social relationships (PSP subscore)	0.8 (0.4)	0.7 (0.5)	0.023	**−**0.07	0.123
Self-care (PSP subscore)	**0.3 (0.5)**	**0.2 (0.4)**	**0.011**	**−0.11**	**0.023**
Disturbing and aggressive behaviors (PSP subscore)	0.1 (0.3)	0.0 (0.1)	<0.001	**−**0.02	0.655
Single	**677 (93.6%)**	**134 (77.5%)**	**<0.001**	**0.38 (0.23; 0.65)**	**<0.001**
Living in her/his own housing	**247 (34.4%)**	**131 (74.4%)**	**<0.001**	**2.85 (1.89; 4.30)**	**<0.001**
Parenthood	**47 (6.4%)**	**42 (23.7%)**	**<0.001**	**2.51 (1.49; 4.22)**	**0.001**
Addictions, *N* (%)					
Current alcohol use disorder	48 (6.5%)	11 (6.2%)	0.891		
Current tobacco use disorder*	400 (56.4%)	83 (48.5%)	0.063	0.83 (0.57; 1.21)	0.340
Current cannabis use disorder*	55 (7.4%)	7 (4.0%)	0.097	0.75 (0.32; 1.79)	0.517
Access to treatments, *N* or Mean (% or SD)					
Second Generation Antipsychotics	558 (75.5%)	144 (81.4%)	0.099	1.38 (0.88; 2.17)	0.166
Cognitive Behavioral Therapy in the last 12 months	28 (3.8%)	10 (5.6%)	0.265		
Antipsychotic daily dose (CPZ-Eq, mg/day)	538.1 (555.2)	586.7 (678.5)	0.379		
Adherence to treatment (MARS total score)	6.1 (2.2)	6.4 (2.2)	0.090	**−**0.01	0.722
Physical health, *N* or Mean (% or SD)					
Total physical activity level (WHO definition)	72.7 (142.0)	21.3 (47.2)	<0.001	0.00	0.996
Body Mass Index (kg/m^2^)	26.2 (5.3)	26.9 (5.2)	0.113	0.01	0.853
Abdominal circumference (cm)	**94.4 (15.3)**	**97.2 (15.2)**	**0.048**	**−**0.01	0.899
History of coronary heart disease	2 (0.3%)	1 (0.6%)	0.475		
High Blood Pressure	**110 (17.2%)**	**18 (11.8%)**	**0.106**	**0.49 (0.27; 0.86)**	**0.014**
LDL-Cholesterol (g/L)	**3.0 (0.9)**	**3.4 (1.0)**	**<0.001**	**0.10**	**0.016**
HDL-Cholesterol (g/L)	1.3 (0.5)	1.3 (0.4)	0.363		
Triglycerides (g/L)	1.5 (1.1)	1.8 (1.6)	0.038	0.03	0.454
Blood glucose (mmol/L)	4.9 (0.9)	5.2 (1.4)	0.003	0.05	0.276
hs-CRP (mg/L)	3.3 (6.7)	3.5 (8.1)	0.745		
Cholecalciferol (nM)	44.8 (25.2)	44.7 (23.7)	0.983		

Poverty is the reference group: a beta >0 or an odds ratio >1 means that the score (or frequence) is lower in the poverty group. Associations are statistically bilateral. Poverty (no vs. yes) is here an explaining variable (independent variable). Multivariate models are therefore adjusted for age, sex and education level and PANSS total score variables for all variables except for those directly related to clinical symptomatology (*). Significant associations are in bold (*p* < 0.05). BAS, Barnes akathisia scale; BIS, Birchwood insight scale; CDSS, Calgary depression scale for schizophrenia; CGI, clinical global impression; CPZ-Eq, chlorpromazine-equivalent; ECPDS, evaluation of cognitive processes involved in disability in schizophrenia; S-QoL, schizophrenia – quality of life; hs-CRP, highly sensitive – C reactive protein; HDL, high density lipoprotein; LDL, low density lipoprotein; MARS, medication adherence rating scale; PANSS, positive and negative syndrome scale; PSP, personal and social performance scale; SAS, Simpson-Angus scale.

**Table 2:** Patient-reported outcomes associated with poverty in schizophrenia.

Variables	Univariate model			Multivariate model	
	Poverty *N* = 739 (80.7%)	No poverty *N* = 177 (19.3%)	Univariate *p* value	Adjusted beta coefficients or adjusted OR	Adjusted *p* value
Patient-reported outcomes					
Quality of life					
S-QoL total score	51.7 (18.7)	50.9 (17.7)	0.610		
Physical well-being (S-QoL subscore)	45.4 (28.1)	46.1 (28.1)	0.758		
Psychological well-being (S-QoL subscore)	53.8 (27.2)	52.8 (27.4)	0.651		
Self-esteem (S-QoL subscore)	**48.7 (29.9)**	**44.0 (28.9)**	**0.061**	**−0.08**	**0.039**
Family relationships (S-QoL subscore)	69.5 (25.9)	67.5 (26.3)	0.382		
Friendships (S-QoL subscore)	48.7 (29.8)	48.5 (27.2)	0.946		
Sentimental life (S-QoL subscore)	34.1 (29.6)	33.6 (31.3)	0.848		
Autonomy (S-QoL subscore)	59.4 (27.5)	57.7 (27.3)	0.483		
Resilience (S-QoL subscore)	54.9 (27.5)	57.1 (25.0)	0.319		
Self-reported aggressiveness (BPAQ scale)					
Verbal aggressiveness (BPAQ subscore)	12.6 (3.9)	12.4 (3.8)	0.521		
Physical aggressiveness (BPAQ subscore)	**20.3 (7.6)**	**17.1 (6.3)**	**<0.001**	**−0.07**	**0.043**
Anger (BPAQ subscore)	17.2 (5.9)	15.9 (5.3)	0.006	**−**0.04	0.353
Hostility (BPAQ subscore)	22.1 (6.7)	22.4 (6.9)	0.653		

Poverty is the reference group: a beta >0 or an odds ratio >1 means that the score (or frequence) is lower in the poverty group. Associations are statistically bilateral. Poverty is here an explaining variable (independent variable). Multivariate models are therefore adjusted for age, sex and education level and PANSS total score. Significant associations are in bold (*p* < 0.05).

In multivariate analyses, patients without poverty demonstrated better clinician-rated outcomes compared to those with poverty across the following variables: lower current psychotic severity (adjusted beta coefficient (aB), aB = −0.12, *p* < 0.001), positive symptoms (aB = −0.10, *p* = 0.007), negative symptoms (aB = −0.11, *p* = 0.002), disorganization (aB = −0.08, *p* = 0.020), excitement (aB = −0.09, *p* = 0.009), and lifetime history of planned suicide (adjusted odds ratio (aOR) = 0.60 [0.41; 0.88], *p* = 0.010). In terms of caregivers-rated outcomes, they exhibited impaired cognitive processes involved in disability (EPHP score) (aB = 0.19, *p* = 0.002), neurocognition (aB = 0.20, *p* = 0.001), motivation (aB = 0.17, *p* = 0.006), insight (aB = 0.13, *p* = 0.046), physical aggressiveness (aB = −0.07, *p* = 0.043), clinical global impression (CGI score) (aB = −0.10, *p* = 0.001), relationship status (singleness) (aOR = 0.38 [0.23; 0.65], *p* < 0.001), independent housing (aOR = 2.85 [1.89; 4.30], *p* < 0.001), parenthood (aOR = 2.51 [1.49; 4.22], *p* = 0.001), unemployment (aOR = 0.11 [0.07, 0.18], *p* < 0.001), social functioning (aB = 0.18, *p* < 0.001), socially useful activities (aB = −0.24, *p* < 0.001), self-care (aB = −0.11, *p* = 0.023), high blood pressure (aOR = 0.49 [0.27, 0.86], *p* = 0.014), and LDL-cholesterol blood levels (aB = 0.10, *p* = 0.016). Conversely, patients with poverty reported higher self-esteem (aB = −0.08, *p* = 0.039) compared to those without poverty. No significant differences were found for addictions, treatments, treatment access, and treatment side effects (all *p* > 0.05).

## Discussion

In the FACE-SZ cohort, which was nationally recruited in a high-income Western country and consisted of stabilized patients, 80% reported monthly incomes below 1,000 euros, indicating poverty. Our investigation confirmed associations between poverty and specific psychiatric outcomes, including more severe psychotic symptoms (though not depressive symptoms), aggressive behavior, singleness, limited opportunity for parenthood and independent housing, as well as impaired professional and social functioning. Moreover, poverty was linked to increased metabolic disturbances such as hypertension and high LDL-cholesterol, despite ongoing psychiatric follow-up and without discernible treatment-related differences. However, poverty did not demonstrate associations with addiction, reduced treatment adherence, decreased access to psychotherapy, or lower insight into illness.

A notable discovery from our study is the marked prevalence of poverty within the FACE-SZ cohort. However, this finding warrants careful interpretation, considering that a majority of the patients receive care within the public sector, potentially leading to the referral of more complex cases to the Expert Center. Patients with higher income might opt for private sector care, potentially contributing to an overestimation of poverty prevalence in schizophrenia within France through the FACE-SZ cohort. Nonetheless, it is worth noting that the FACE-SZ cohort effectively represents middle-aged stabilized outpatients with schizophrenia in terms of education level, age at illness onset, and comorbidities (31, 32). Importantly, although the FACE-SZ population may not provide a complete representation of the entire French population, it is crucial to note that the expert centers were not exclusively designed to support the impoverished population; rather, they were established to address the needs of all patients with diagnoses or therapeutic concerns.

The anticipated associations between poverty and low education level, unemployment, reduced engagement in socially beneficial activities, being single, and not having children were confirmed. However, it is important to highlight that poverty was not linked to decreased social functioning, heightened depression, or impaired quality of life, contrary to initial expectations. Surprisingly, individuals in poverty reported higher self-esteem levels compared to their wealthier counterparts.

The associations of poverty and biological disturbances has been described in the general population. Metabolic syndrome health disparities are believed to originate in childhood, where various factors such as genetic predisposition, early life environment, and lifestyle habits may interact to influence their development and persistence over time (33). Challenging socioeconomic environments with low resources may hinder the accumulation of a reservoir of assets, referred to as a reserve capacity, and may also induce stress, thereby depleting the existing reserves (34). The elevated risk of cardiometabolic issues in individuals with schizophrenia is often due to a combination of factors, including lifestyle factors such as poor diet and physical inactivity, medication side effects, and the physiological impact of the illness itself (35).

Despite the well-established connection between poverty and an elevated risk of depression in the general population (36), our analysis did not uncover a similar correlation among the participants in our study. However, poverty was found to be correlated with a greater severity of psychotic symptoms. This finding diverges from recent research that reported a moderate association between subjective well-being and socioeconomic status in a meta-analysis of population-based studies (37).

In essence, the relationships between socio-economic status and self-esteem appear distinct in schizophrenia when compared to the general population. Firstly, this finding prompts us to reconsider the traditional assumptions about the relationship between socioeconomic factors and self-esteem. While poverty-related disempowerment might be expected to erode self-esteem, our results suggest a more complex interplay between socioeconomic status and self-perception. Delving into the underlying mechanisms that drive this counterintuitive link could yield valuable insights into how individuals with schizophrenia navigate their sense of self-worth within challenging circumstances.

Moreover, understanding why this specific association exists could have implications for designing targeted interventions. If poverty-related disempowerment is indeed tied to higher self-esteem, interventions could potentially leverage this dynamic to foster better mental well-being and quality of life among individuals facing schizophrenia and poverty. By addressing the factors that contribute to this unexpected relationship, we might uncover new avenues for promoting positive psychological outcomes in this vulnerable population. Individuals who report higher earnings are employed and, as a result, are more exposed to workplace or societal stigma in general.

Lastly, this finding underscores the importance of considering nuanced perspectives in mental health research. Schizophrenia is a complex condition with multifaceted interactions between biological, psychological, and social factors. By exploring the intricate interplay between poverty, self-esteem, and other variables, we contribute to a more comprehensive understanding of the lived experiences of individuals with schizophrenia.

Several explanations can be postulated to elucidate the disparities observed between individuals with schizophrenia and other economically disadvantaged populations. Firstly, we utilized an extremely low threshold to delineate the poverty group. Those earning between 1,000 and 1,500 euros may still grapple with financial challenges pertaining to housing and daily necessities, potentially relying on family members for financial support. This subset of individuals might be employed, encountering workplace stigma that could impact their quality of life. Our findings of diminished self-esteem among those with higher incomes align with this notion. Notably, the reduction of self-stigma only transpires when individuals are not subjected to discrimination within their employment environments (38). Socially useful activities hold significance only when they offer positive personal experiences (39, 40). The “insight paradox” (better insight impacting self-esteem) (41) is not the most plausible explanation, as we observed no significant difference in insight between the poverty and non-poverty groups.

Likewise, no disparity between the groups was found in terms of addictions, treatments, and treatment side effects, implying that France’s comprehensive 100% health insurance coverage effectively mitigates healthcare inequalities. Hence, treatments and addictions are unlikely to account for these differences.

The heightened prevalence of depression in individuals with schizophrenia, three times greater than in the general population (42), could elucidate why both groups exhibit comparable levels of depression. Depression in schizophrenia might be influenced by distinct etiological factors than those in the general population, with a heightened role of biological factors and reduced influence of socio-environmental factors.

It’s important to emphasize that patients facing poverty exhibited no variations in treatments’ access (pharmacological treatment or psychotherapy). In France, treatments for schizophrenia are fully reimbursed, and psychotherapy can be accessed freely within the realm of public mental health facilities.

Indeed, the observation that individuals with schizophrenia constitute a distinct impoverished population in terms of health-related outcomes and social integration has important implications for public health programs. To expand on this, we can identify several specific areas where our findings can guide targeted interventions:

- Tailored Support Services: given the unique challenges faced by individuals with schizophrenia in terms of health outcomes and social inclusion, public health programs could develop tailored support services. These services might encompass specialized mental health interventions that address the nuanced needs of this population, while also considering the influence of poverty-related disempowerment on self-esteem.
- Employment and Stigma Mitigation: as we discussed earlier, individuals reporting higher incomes might experience increased exposure to stigma, especially in work settings. Building on this insight, public health programs could focus on providing job-related support, anti-stigma campaigns, and workplace accommodations to minimize the potential negative impact of stigma on both self-esteem and mental well-being.
- Comprehensive Mental Health Care: our study underscores the complex relationship between poverty, mental health, and social functioning. Public health initiatives could concentrate on ensuring comprehensive mental health care that not only addresses clinical symptoms but also recognizes the broader socioeconomic factors influencing the lives of individuals with schizophrenia.
- Holistic Interventions: by acknowledging the paradoxical association between poverty-related disempowerment and self-esteem, public health programs can adopt a more holistic approach to intervention. This might involve integrating social empowerment strategies into mental health care, aiming to boost self-esteem while addressing the unique challenges posed by poverty.
- Collaboration and Advocacy: engaging stakeholders across multiple sectors, including mental health, social services, employment, and education, can enhance the impact of public health programs. Collaborative efforts and advocacy campaigns can work towards destigmatization, improved access to resources, and fostering social inclusion for individuals with schizophrenia.

### Limits

In this study, poverty was considered as a circumstance preceding the expert center evaluation. We opted to examine factors associated with poverty at enrollment in an initial cross-sectional investigation. A trajectory study, delving into the impact of poverty on FACE-SZ follow-up trajectories, is planned for the future, contingent on the accrual of sufficient follow-up data in the FACE-SZ dataset.

The FACE-SZ cohort did not encompass the participants’ early socioeconomic circumstances, including parental income during childhood, number of siblings, monoparental households, and the current financial status of the patients’ caregivers. The lifelong progression of symptomatology, particularly persistent primary negative symptoms that might influence functional trajectories, could not be captured due to memory biases and the challenges of retrospectively exploring negative symptoms. Our findings were adjusted by factoring in education level, often considered a surrogate for family socioeconomic status.

### Strengths

The adjustment for age, sex, education level, and psychotic symptomatology represents a robust aspect of the current findings. Consequently, the relationships between poverty and functioning cannot be entirely attributed to sociodemographic factors or illness severity. The validity of our results is fortified by the comprehensive national-level recruitment across numerous centers, yielding a substantial sample size. It is worth noting that the correlation between poverty and unfavorable clinical outcomes is unlikely to be solely explicable by limited opportunity for studies or merely by heightened symptom severity.

## Conclusion

More than 8 out of 10 schizophrenia patients live below the poverty threshold. Despite equitable access to care, individuals experiencing mental and physical health challenges continue to fare worse, with limited opportunity for social roles although not to social functioning. Paradoxically, poverty-related disempowerment might be linked to elevated self-esteem. Thus, schizophrenia emerges as a distinct population grappling with health-related outcomes and social integration, warranting attention in public health initiatives.

## Data availability statement

The raw data will be made available on reasonable to the authors.

## Ethics statement

The studies involving humans were approved by CPP-Ile de France IX, January 18th, 2010. The studies were conducted in accordance with the local legislation and institutional requirements. The participants provided their written informed consent to participate in this study.

## Author contributions

LB and GF: concept and design, interpretation of data, drafting of the manuscript, and supervision. GF, DY, BT, JM, MU, SL, RR, DM, P-ML, FS, FB, and LB: acquisition and critical revision of the manuscript for important intellectual content. LB: statistical analysis. All authors contributed to the article and approved the submitted version.

## Footnotes

^1^ www.fondation-fondamental.org
